# Effect of the Titanium Isopropoxide:Acetylacetone Molar Ratio on the Photocatalytic Activity of TiO_2_ Thin Films

**DOI:** 10.3390/molecules24234326

**Published:** 2019-11-27

**Authors:** Jekaterina Spiridonova, Atanas Katerski, Mati Danilson, Marina Krichevskaya, Malle Krunks, Ilona Oja Acik

**Affiliations:** 1 Laboratory of Thin Films Chemical Technologies, Department of Materials and Environmental Technology, Tallinn University of Technology, Ehitajate tee 5, 19086 Tallinn, Estonia; atanas.katerski@taltech.ee (A.K.); malle.krunks@taltech.ee (M.K.); 2 Laboratory of Optoelectronic Materials Physics, Department of Materials and Environmental Technology, Tallinn University of Technology, Ehitajate tee 5, 19086 Tallinn, Estonia; mati.danilson@taltech.ee; 3 Laboratory of Environmental Technology, Department of Materials and Environmental Technology, Tallinn University of Technology, Ehitajate tee 5, 19086 Tallinn, Estonia

**Keywords:** TiO_2_, thin film, ultrasonic spray pyrolysis, acetylacetone, photocatalysis, stearic acid

## Abstract

TiO_2_ thin films with different titanium isopropoxide (TTIP):acetylacetone (AcacH) molar ratios in solution were prepared by the chemical spray pyrolysis method. The TTIP:AcacH molar ratio in spray solution varied from 1:3 to 1:20. TiO_2_ films were deposited onto the glass substrates at 350 °C and heat-treated at 500 °C. The morphology, structure, surface chemical composition, and photocatalytic activity of the obtained TiO_2_ films were investigated. TiO_2_ films showed a transparency of ca 80% in the visible spectral region and a band gap of ca 3.4 eV irrespective of the TTIP:AcacH molar ratio in the spray solution. TiO_2_ films consist of the anatase crystalline phase with a mean crystallite size in the range of 30–40 nm. Self-cleaning properties of the films were estimated using the stearic acid (SA) test. A thin layer of 8.8-mM SA solution was spin-coated onto the TiO_2_ film. The degradation rate of SA as a function of irradiation time was monitored by Fourier-transform infrared spectroscopy (FTIR). An increase in the TTIP:AcacH molar ratio from 1:4 to 1:8 resulted in a ten-fold increase in the photodegradation reaction rate constant (from 0.02 to the 0.2 min^−1^) under ultraviolet light and in a four-fold increase under visible light.

## 1. Introduction

The synthesis and optimization of TiO_2_ thin films is a rapidly developing topic in the field of environmental engineering due to their great potential in pollution treatment and self-cleaning properties. Transparent glass has multiple applications. It is widely used in our homes (in windows, doors, and shower enclosures), in automobile windshields, in electronic device screens, and in construction materials for modern skyscrapers. Keeping the surface of the glass clean is not an easy task; due to its optical transparency, all the dirt particles, stains, and fingerprints which present on the surface are noticeable [1].

Today, more and more skyscrapers are built worldwide because they give us an opportunity to design a good deal of real estate using a relatively small ground area. However, skyscraper window cleaning is risky. According to the National Institute for Occupational Safety and Health (NIOSH) in the United States there were 88 window cleaning accidents over a 15-year period; 70% of them ended in death [2]. Covering the surface of skyscrapers with a TiO_2_ thin layer can substitute this risky job and save money (along with water and chemicals) on cleaning. TiO_2_ has photocatalytic and hydrophilic properties when it is irradiated with light source that has an energy content equal to or higher than the band gap of the TiO_2_. One of the first commercialized applications of TiO_2_ as a photocatalytic material was a self-cleaning glass for tunnel light covers in Japan. Tunnel lamps in Japan emit ultraviolet (UV) light of about 3 mW cm^−2^, which is enough to keep the glass cover surface clean of automobile exhaust when it is covered with the TiO_2_ photocatalyst [3].

For commercial use, it is very important to find a cost-effective and efficient technology for thin film deposition. TiO_2_ films have been prepared using a variety of deposition methods such as chemical vapor deposition [4], sputtering [5], hydrothermal [6], electrophoretic [7], sol-gel [8,9], and spray pyrolysis [10,11]. Among the above-mentioned methods, deposition of thin films with ultrasonic spray pyrolysis has the advantage of being a simple, inexpensive method that allows us to obtain transparent homogeneous films with high stability and quality. This method could be easily applied at the laboratory scale as well as in industry for film deposition on large surfaces [12,13]. Our research group is focused on gas-phase photocatalytic oxidation on TiO_2_ thin films prepared by the spray pyrolysis technique. In our recent study, 350 °C was found as to be an optimal temperature for film deposition because this film has a greater number of surface defects [13].

Choosing a precursor solution for thin film preparation is the next essential step in the process of material synthesis. In solution preparation, mainly alkoxides are used as a titanium source, for example, titanium butoxide [12,14], titanium ethoxide [15], and titanium isopropoxide (TTIP) [8,13,16]. Since metal oxides are highly reactive with water, to stabilize the precursor solution and retard the hydrolysis rate, chemical additives are used. These can be acids (HCl) [17], bases (NaOH) [18], solvents (2-methoxyethanol) [9,19], or stabilizing agents (acetylacetone) [9,10,16]. The effects of organic additives such as benzoic acid [20], diethylene glycol [21], diethanolamine [22], humic acid [23], and glucose [24] on the properties of TiO_2_ thin films and powder materials were studied. Byrne et al. (2016) have studied the effect of molar ratio TiO_2_:benzoic acid on the phase transformation and carbon incorporation onto the surface of photocatalyst and found that benzoic acid enhances rutile phase formation [20]. Park and co-authors (2009) studied the carbon incorporation into the lattice of TiO_2_ photocatalyst prepared by sol-gel by changing the calcination temperature without the addition of external carbon source. Lattice carbon incorporation enhances the photocatalytic oxidation of 4-chlorophenol and iodide under visible light [25].

In the current study, modifications in the precursor solution were done to prepare a more active material. Titanium isopropoxide (TTIP) was used as a precursor and acetylacetone (AcacH) as a stabilizing agent. The reaction of TTIP with AcacH has been well studied in the literature. Acetylacetone behaves as a nucleophilic reactant and replaces the alkoxy group; thus, a new molecular precursor is formed [26]. This reaction has been studied using Fourier-transform infrared spectroscopy (FTIR) [16,27]. However, it is known that the TTIP:AcacH molar ratio 1:1 is not sufficient for complete complex formation; the complex is formed at the molar ratio of 1:2 and starting from the ratio 1:3 an excess of acetylacetone can be seen in the solution [16]. Few studies have focused on the effect of the TTIP:AcacH molar ratio on the structural properties [16] and photocatalytic activity of the films [28] and powders [29]. No comprehensive studies about addition of a high AcacH molar ratio into the precursor solution and its effects on the material and photocatalytic properties of the films were found in the scientific literature. The aim of this study was to determine the optimal TTIP:AcacH molar ratio in the precursor solution for the deposition of photocatalytically active TiO_2_ thin films by the ultrasonic spray pyrolysis method. The morphological and structural properties, surface chemical composition, and photocatalytic activity of TiO_2_ thin films with different TTIP:AcacH molar ratios (from 1:3 to 1:20) were investigated. The photocatalytic activity in the current study was tested by the stearic acid test under ultraviolet (UV-A) and visible light (VIS).

## 2. Results and Discussion

### 2.1. Surface Morphology

**Figure 1:**
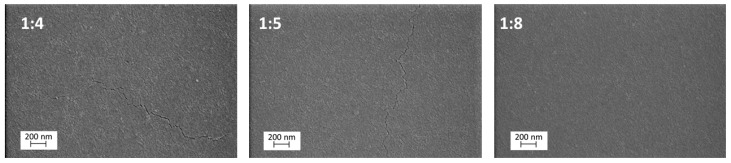
Scanning electron microscopy (SEM) surface images of TiO_2_ thin films deposited on borosilicate glass at titanium isopropoxide (TTIP):acetylacetone (AcacH) molar ratios of 1:4, 1:5, and 1:8.

Scanning electron microscopy (SEM) images of the TiO_2_ thin films with TTIP:AcacH molar ratios of 1:4, 1:5, and 1:8 are presented in Figure 1. An increase in the amount of acetylacetone in the precursor solution makes the surface of the thin film flatter, smoother, and more uniform. Kajitvichyanukul et al. (2005) have studied the effect of acetylacetone on the morphology of TiO_2_ thin films prepared by sol-gel dip coating. They found that with the increase in acetylacetone, smooth films, like TiO_2_ sheets, were obtained [30].

Figure 1 shows that the surfaces of 1:4 and 1:5 TTIP:AcacH thin films have cracks, while the 1:8 film surface is crack-free. Addition of acetylacetone to titanium isopropoxide makes the process of gel formation slower. Therefore, the precursor solution is more stable and homogeneous, promoting the synthesis of thin films with a higher quality and better adhesion [26].

**Figure 2:**
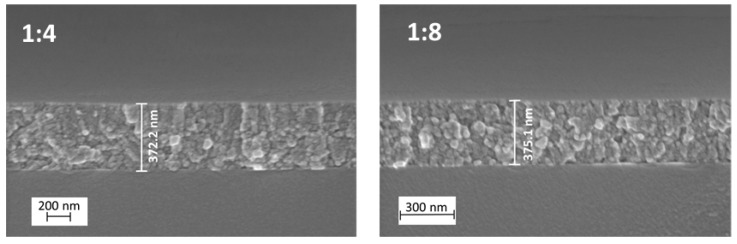
Cross-sectional SEM images of TiO_2_ thin films deposited on borosilicate glass at TTIP:AcacH molar ratios of 1:4 and 1:8.

The thicknesses of the samples with the molar ratios 1:4 and 1:8 were determined from their SEM cross-sectional images and were 370 and 375 nm, respectively (Figure 2). It was observed that the thickness of the TiO_2_ thin films was not affected by the increase in the acetyalacetone molar ratio in the starting solution.

### 2.2. Structural Properties

**Figure 3:**
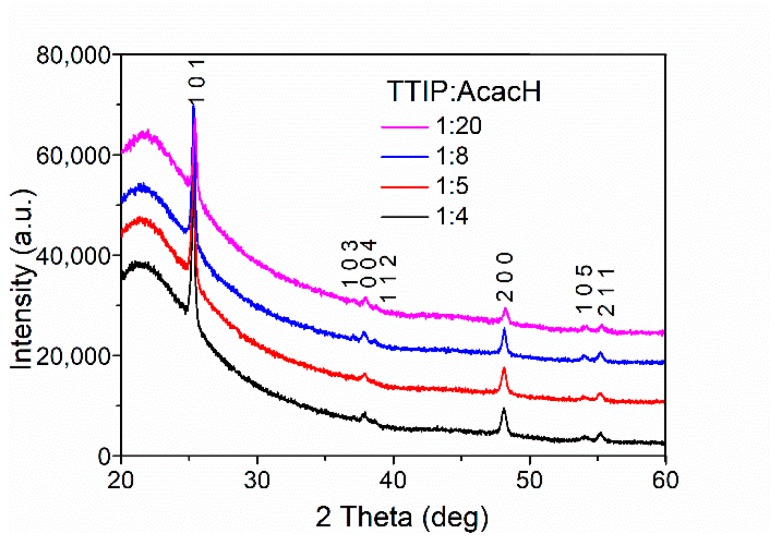
X-ray diffraction (XRD) patterns of TiO_2_ thin films with different TTIP:AcacH molar ratios.

X-ray diffraction (XRD) analysis was performed to investigate crystallinity, phase composition, and the mean crystallite size of TiO_2_ thin films. All of the obtained films consist only of the anatase phase regardless of the acetyalacetone molar ratio in the precursor solution. Figure 3 reveals a difractogram of TiO_2_ films. The diffraction peaks at 2 theta of 25.3°, 37,0°, 37.8°, 38.6°, 48.1°, 53.9°, and 55° correspond to the reflection planes from (101), (103), (004), (112), (200), (105), and (211), respectively, of anatase TiO_2_ [31]. No diffraction peaks belonging to the rutile or brookite phase were detected.

The mean crystallite size was calculated using the full width at half maximum (FHWM) of the most intense (101) peak of anatase phase by the Scherrer formula. The mean crystallite size of the films varies in the range of 30–40 nm. Slight changes in the values of crystallite size are not in correlation with the amount of acetylacetone in the starting solution.

According to the literature, an increase of AcacH in the precursor solution affects the phase transformation process from anatase to rutile and enhances the crystallization process. The anatase to rutile phase transformation starts to occur at the annealing temperature about 800 °C when the precursor solution is not fully stabilized (in the case of a TTIP:AcacH molar ratio less than 1:3). Excess of AcacH in the starting solution lowers the transformation temperature by about 100 °C [16].

The results of this study demonstrate that an increase in the amount of the unchelated acetylacetone in the precursor solution has no significant effect on the structural properties of TiO_2_ thin films, since all the obtained films were annealed at the same temperature −500 °C.

### 2.3. Optical Properties

**Figure 4:**
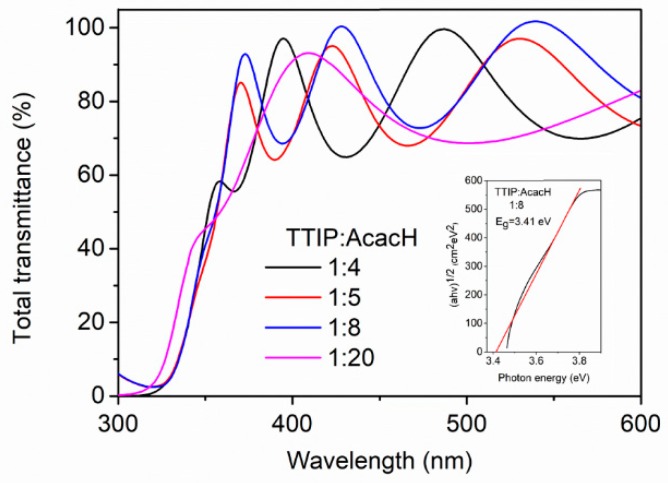
Total transmittance spectra for TiO_2_ thin films deposited at different TTIP:AcacH molar ratios. The inset shows the band gap value of the sample deposited at the TTIP:AcacH molar ratio of 1:8.

The optical transmittance spectra of TiO_2_ thin films were measured in the wavelength range between 200 and 1200 nm (Figure 4). Obtained thin films are transparent in the visible light region. The transmittance is about 80% in the spectral region of 500–1200 nm for all obtained films irrespective of the TTIP:AcacH molar ratio in the spray solution.

Using maxima or/and minima of interference fringes of the optical spectrum, it is possible to investigate the thickness of the film by the so called Swanepoel’s method. Thickness (*d*) can be found by the following equation:

(1) $$d=\frac{\lambda_{1}\lambda_{2}}{2\left(\lambda_{1}n_{2}-\lambda_{2}n_{1}\right)},$$

where n_1_ and n_2_ are reflective indexes at two adjacent maxima/minima, and $\lambda_{1}\lambda_{2}$ is the wavelength of the maxima/minima [32].

The thickness calculated by Swanepoel’s method corresponds to the thickness that was determined by SEM cross-sectional images (Figure 2, Section 2.1.) and is about 400 nm for all samples (Table 1), except for the sample with the TTIP:AcacH molar ratio of 1:20. This sample is about 100 nm thinner. This could be explained by a rise in the amount of free AcacH in precursor solution that increased the viscosity of the spray solution. Viscosity affects the transportation of aerosol produced by an ultrasonic generator. The aerosol flow rate decreases with the increase in the viscosity value. As a result, the growth of the thin film occurs more slowly [33].

The band gap was found using the Tauc plot. Since TiO_2_ has an indirect band gap, a plot of the $\sqrt{\alpha E}$ function of photon energy, E = hυ, was used [34]. Table 1 shows the band gaps of the obtained TiO_2_ thin films, which are about 3.4 eV.

Overall, it was found that the optical properties of the TiO_2_ films are not significantly affected by the acetylacetone amount in the precursor solution.

### 2.4. Chemical Composition and Wettability

**Figure 5:**
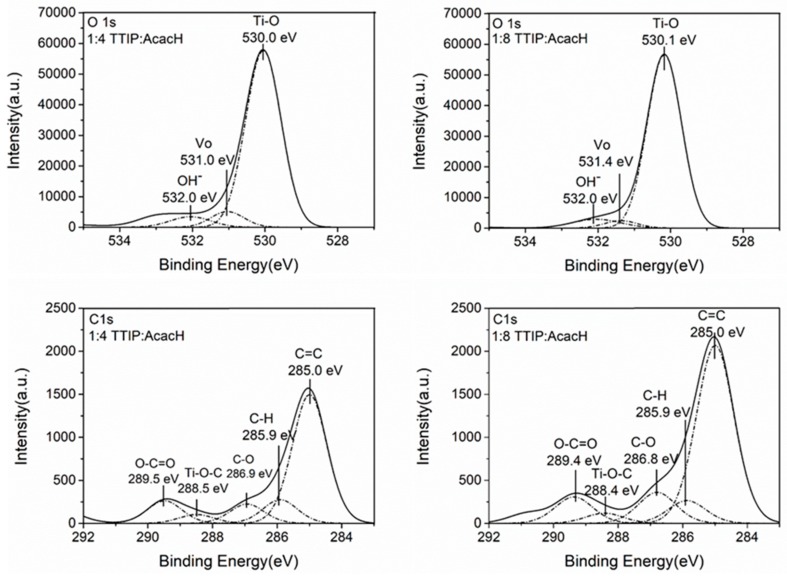
X-ray photoelectron spectroscopy (XPS) spectra of C_1s_ and O_1s_ core levels for 1:4 and 1:8 TTIP:AcacH molar ratios for TiO_2_ thin films after ultraviolet (UV-A) pre-treatment.

X-ray photoelectron spectroscopy (XPS) study was performed to investigate the chemical composition and bonding structure of the obtained TiO_2_ thin films. The XPS spectra of TiO_2_ thin films after UV-A pre-treatment for O_1s_ and C_1s_ core levels are represented in Figure 5.

O_1s_ core level spectra consist of three peaks (Figure 5). The highest peak at the binding energy (BE) value 530.0 eV corresponds to the Ti-O bond. The peak at the BE value 531.0 eV indicates the O^2−^ ions in oxygen-deficient regions within the matrix of TiO_2_ [35], which could be associated with oxygen vacancies (V_o_), and the peak at the BE value 532.0 eV corresponds to hydroxyl groups adsorbed on the surface [36,37].

The carbon region consists of five singlets with maxima located at 285.0 eV, 285.9 eV, 286.9 eV, 288.5 eV, and 289.5 eV. The highest peak located at 285.0 eV originates from the C=C bond. The peak with the BE maxima at 285.9 eV corresponds to surface hydrocarbons. These forms of non-oxygenated carbons belong to contaminants adsorbed on the TiO_2_ surface. Some studies propose that this carbon may exist in the grain boundaries of TiO_2_ and it therefore acts as a photosensitizer [37,38]. Peaks with the BE values 286.9 eV and 288.5 eV represent the oxygen-bound species C-O and Ti-O-C bonds, respectively [39]. These peaks confirm the incorporation of carbon into the lattice in the place of the Ti atoms, i.e., interstitial carbon doping takes place [38].

The integrated areas of the peaks were used to calculate atomic concentrations of the chemical components. The atomic ratios of the components (OH)/(Ti–O), (Vo)/(Ti–O), (C-O)/(C=O) and (C =C)/(C=O) ratios are presented in Table 2. There is no significant difference in the amount of oxygen vacancies and hydroxyl groups with the increase in AcacH, since the deposition parameters were the same. In their study, Dündar et al. (2019) showed that oxygen defects and the hydroxyl group amount on the surface depend on the temperature of the deposition [13].

XPS results showed that films with the higher AcacH molar ratio of 1:8 contained more adsorbed carbon on the surface of the film than films with TTIP:AcacH molar ratio of 1:4. It indicates that the surface of the 1:8 film could be more active since during the photocatalytic experiments, the oxidation of surface contaminant carbon takes place that leads to the increased number of active sites on the surface of the thin film [40]. The amount of C-O and Ti-O-C at the molar ratio 1:8 is also higher, which means that carbon from the unchelated acetylacetone is transported to the TiO_2_ lattice during the decomposition of organic matter. It is reported in the literature that carbon doping enhances photocatalytic activity since it results in localized states occupied in the band gap [41,42]. One carbon atom incorporation into the TiO_2_ lattice in an interstitial position generates three strong covalent C-O bonds. These bonds in turn, create three bonding C-O states below the bottom of the O_2p_ valence band. Corresponding antibonding C-O states with high energy lie in the conduction band. The carbon impurity in the interstitial position leads to the excess of electrons. Excess electrons moves from C-O state to the Ti_3d_, not to corresponding C-O antibonding states, which creates two new occupied states in the band gap, Ti_3d_ and C_2p_, below the bottom of the conduction band [43].

The wettability of the TiO_2_ film surface was measured for as-deposited films after thermal treatment, one-month aged films, and for aged films after 15 min of UV-A pre-treatment. The average water contact angle (CA) values are shown in Table 3. There is no difference in wettability for as prepared samples with an increase of AcacH in precursor solution; both surfaces are hydrophilic with a CA of about 20°. After one month of storage in the plastic box hydrophilic properties of the films decreased while the sample with a lower AcacH molar ratio (1:4) had significantly higher CA. Contact angles increased to 55° and 30° for TTIP:AcacH molar ratios of 1:4 and 1:8, respectively. This means that the increase of acetylacetone in the precursor solution helps to keep the surface of the films photoinduced for a longer period of time, i.e., adsorption of water and contaminants from the ambient air on the surface of the TiO_2_ film with a TTIP:AcacH molar ratio of 1:8 occurs more slowly than at a ratio of 1:4. [44,45,46]. However, after 15 min of UV-A pre-treatment, all samples became super-hydrophilic, with CA less than 5°.

During the UV-A pre-treatment water molecules desorb from TiO_2_ surface and hydroxyl groups are formed, leading to a better wetting of the surface [46]. This is in accordance with the XPS results showing that after UV-A pre-treatment, the number of hydroxyl groups on the TiO_2_ surface is the same for TTIP:AcacH molar ratios of 1:4 and 1:8 (Table 2). During the UV-A pre-treatment, besides hydroxyl groups, peroxo species (H_2_O_2_) are formed on the surface of the photocatalyst, which increases the photocatalytic activity of the material [47,48]. Organic substances on the surface of TiO_2_ act as electron donors and enhance the production of H_2_O_2_ [47].

### 2.5. Photocatalytic Activity. Stearic Acid Test

The stearic acid (SA) test is a method for assessing the activity of self-cleaning photocatalyst films. The essence of this method is a deposition of a thin layer of SA onto the film of photocatalytic material and monitoring of the the destruction of SA as a function of irradiation time. The overall reaction can be summarized as follows:

$${{\mathrm{CH}}_{3}({\mathrm{CH}}_{2})}_{16}{\mathrm{CO}}_{3}H+26O_{2}\overset{\begin{array}{c} TiO_{2}\\ hv\geq Eg \end{array}}{\to }18{\mathrm{CO}}_{2}+18H_{2}O$$

**Figure 6:**
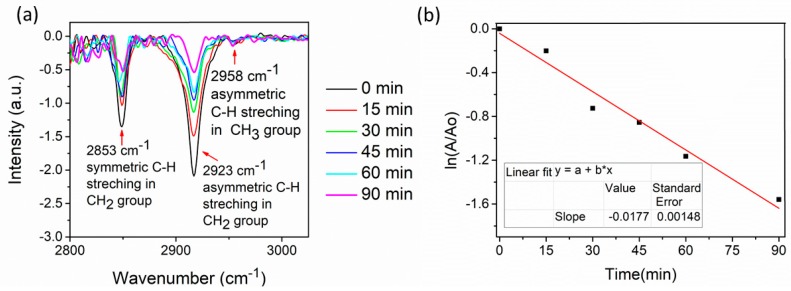
Fourier-transform infrared spectroscopy (FTIR) spectra of stearic acid on the surface of TiO_2_ at the TTIP:AcacH molar ratio of 1:4 (**a**) and determination of the photodegradation rate constant (k) at the molar ratio of 1:4 (**b**).

The most commonly employed method of monitoring photodegradation of stearic acid is via the disappearance of the SA film using infrared absorption spectroscopy. SA absorbs strongly in the region 2700–3000 cm^−1^, with peaks at 2958 cm^−1^, 2923 cm^−1^, and 2853 cm^−1^ due to asymmetric in-plane C-H stretching in the CH_3_ group and asymmetric and symmetric C-H stretching in the CH_2_ groups, respectively (Figure 6a) [49,50,51].

A stearic acid layer was deposited after the TiO_2_ film was pre-treated under UV-A to make the surface of the film photo-induced and super-hydrophilic. The decrease in stearic acid bond intensity was observed by FTIR measurements as a function of irradiation time. FTIR spectra were measured before the UV-A/VIS irradiation and every 15 min during an hour. The integrated area of the band was used to measure the degradation of stearic acid. It has been observed by several authors [52] that the surface structural and wettability properties of the films affect the growth of SA layer on the surface of a photocatalytic material. Smirnova et al. (2015) detected a serious difference in the SA peaks intensities for TiO_2_ thin films annealed at different temperatures [52]. In this study, films have similar structural and wettability properties (Section 2.2 and Section 2.4); therefore, the initial integrated area of SA is almost the same.

Degradation of SA is known to follow first-order kinetics, i.e., the integrated band area (A) depends on time as follows:

A(t) = A_0_e^−kt^, (2)

where k is photodegradation rate constant. The photodegradation rate constant is the slope value of the linear fit of the plot ln(A/A_o_) versus t [53]. The degradation of stearic acid by TiO_2_ at the TTIP:AcacH molar ratio 1:4 and determination of the photodegradation rate constant are presented in Figure 6.

**Figure 7:**
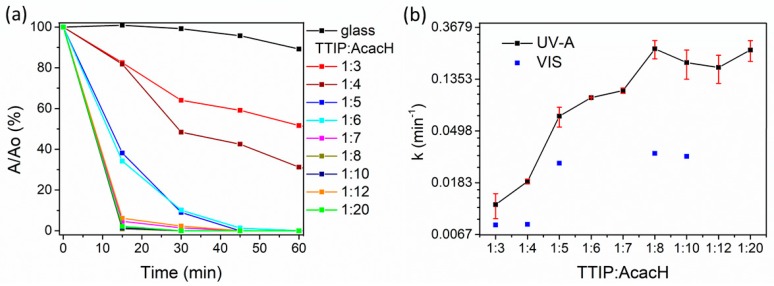
The degradation of stearic acid as a function of ultraviolet (UV-A) irradiation time (**a**) and photodegradation rate constants (k) under UV-A and visible (VIS) light (**b**) for TiO_2_ thin films at different TTIP:AcacH molar ratios.

The degradation of the stearic acid layer as a function of UV-A irradiation time and the photodegradation rate constants (k) at different TTIP:AcacH molar ratios under UV-A and VIS light for obtained TiO_2_ thin films are illustrated in Figure 7. 

Figure 7 shows that the increase in acetylacetone in the precursor solution results in a rapid increase in the photodegradation rate of SA. As can be seen in Figure 7a, for the TTIP:AcacH molar ratios of 1:3 and 1:4, 60 min of UV-A irradiation were not sufficient for complete degradation of the 8.8-mM SA layer; the degradation values were 48% and 69%, respectively. For the molar ratios of 1:5 and 1:6, the degradation was about 90% after 30 min, while at the higher ratios 1:7–1:20, almost complete degradation was achieved after 15 min of UV-A irradiation.

Pore et al. (2006) achieved complete degradation of the 8.8-mM SA layer degradation for atomic layer-deposited TiO_2_ thin films modified with H_2_S after 60 min of UV-A irradiation, while an unmodified TiO_2_ film was capable of degrading only half of SA at the same time of irradiation [51]. The optimal TTIP:AcacH molar ratio found was 1:8 at the reaction rate constant value 0.2427 min^−1^. As Figure 7b shows, after the molar ratio 1:8, the precursor solution is saturated with AcacH. The plateau is formed and no more increase in the photodegradation rate occurs.

According to many authors, carbon doping enhances photocatalytic activity under visible light [25,54,55,56,57,58,59]. Therefore, some of the films were tested for the ability to degrade the stearic acid layer under VIS light (Figure 7b). Photodegradation rate constants under VIS light are on average five times lower than under UV-A light. However, the trend in the increase of k value with the increase in amount of AcacH is similar to the case of UV-A. Films were not capable of full mineralization of the SA layer after 60 min of VIS irradiation. However, the conversion of SA was about 90% for samples at 1:8 and 1:10. Ratova et al. (2018) found that interstitial carbon doping enhances the photocatalytic activity of the reactively co-sputtered TiO_2_ films under UV and VIS light [55]. However, the red shift of the band gap to the visible spectra was not observed in the present study (Table 1). The enhanced photocatalytic activity of the films under UV-A caused by an increase of AcacH can be due to the formation of C-O bonds, which could form new occupied states in the band gap (Section 2.4) [43].

## 3. Materials and Methods

### 3.1. TiO_2_ Thin Film Synthesis

TiO_2_ thin films were deposited onto borosilicate glass substrates by the ultrasonic spray pyrolysis technique. Titanium (IV) isopropoxide (TTIP) was used as a titanium source, acetylacetone (AcacH) as a stabilizing agent, and ethanol as a solvent. All the deposition parameters were kept constant except the amount of acetylacetone in the precursor solution. The molar ratio of TTIP:AcacH in the precursor solution varied from 1:3 to 1:20. The TTIP concentration in the precursor solution was 0.2 M. To prepare the precursor solutions different amounts of acetylacetone were added to the 3.5 mL TTIP and after some minutes of mixing, the solution was adjusted to the 60 mL by ethanol. Prepared solutions were stirred for one hour. Aerosol produced by an ultrasonic generator was transported by compressed air with a flow rate of 5 L/min. The deposition temperature was set up to 350 °C. As-deposed samples were annealed at 500 °C for 1 h in air.

### 3.2. Characterization of Films

Scanning electron microscope (SEM, Carl Zeiss, Jena, Germany) was used to investigate the surface morphology of the samples and cross-section images were used to determine the thickness of the films. X-ray diffraction (XRD) was used to study structural properties of the obtained TiO_2_ thin films. The XRD diffractograms were obtained from the Rigaku Ultima IV diffractometer (Tokyo, Japan) with Cu Kα radiation of λ = 1.5406 Å, 40 kV, at 40 mA, using a silicon strip detector. The measurements were performed in 2 theta configurations with the scan range 20–60°, with a scanning speed rate of 5° min^−1^ and a step of 0.02°. The mean crystallite size was calculated using the Scherrer formula from the full width at half maximum (FWHM) of the most intense peak (101) reflection of TiO_2_ anatase phase. Optical properties were measured with Jasco V-670 UV-VIS-NIR spectrophotometer (Tokyo, Japan) equipped with an integrating sphere in the spectral range 250–1500 nm. X-ray photoelectron spectroscopy (XPS) with use of a Kratos Analytical AXIS ULTRA DLD spectrometer (Manchester, England) in conjunction with a 165-mm hemispherical electron energy analyzer (Kratos Analytical, Manchester, England) and delay-line detector (Kratos Analytical, Manchester, England) was used to investigate the chemical composition of the surface of TiO_2_ thin films. The analysis was carried out with monochromatic Al Kα X-rays (1486.6 eV) operating at 15 kV and 150 W. All XPS spectra were recorded using an aperture slot of 300–700 mm and pass energy of 20 eV. Binding energy (BE) values were calculated based on the C1s core level peak at 285.0 eV. Wettability of the films was studied with water contact angle (CA) measurements.

### 3.3. Evaluation of Photocatalytic Activity

Photocatalytic activity of the thin films was studied using a stearic acid (SA) test. A thin layer of 8.8-mM SA solution was deposited onto the TiO_2_ thin film by the spin coating technique. For this, 100 µL of SA solution in methanol was dropped onto the center of the film (dimensions of the sample ≈ 2 × 2 cm). The rotation speed was 1000 rpm and the rotation time was 30 s. After the deposition, samples with stearic acid layer were dried at 80 °C in air for 10 min. The degradation of SA as a function of UV-A or VIS irradiation time was monitored by Fourier-transform infrared spectroscopy (FTIR, PerkinElmer, Beaconsfield, England) in the wavenumber region 3200–2500 cm^−1^ in a transmission mode [46,47]. The instrument parameters for the measurements were as follows: wavenumber region 3200–2500 cm^−1^, number of scans 32, resolution 4 cm^−1^. The UV Philips Actinic BL 15 W (Philips, Poland), irradiance 3.5 mW cm^−2^ with reflector (integrated in the range of 180–400 nm, with maximum emission at 365 nm, UV-B/UV-A ratio < 0.2%) or the VIS Philips TL-D 15 W (Philips, Poland), irradiance 3.3 mW cm^−2^ with reflector lamps (integrated in the range of 180–700 nm, UV/UV-VIS ratio < 5%) were used as an irradiation source. The sample of the TiO_2_ thin film without a stearic acid layer on it was used as a background during all the measurements. The integrated area of the bands was used to measure the degradation of stearic acid.

## 4. Conclusions

TiO_2_ thin films with TTIP:AcacH molar ratios from 1:3 to 1:20 were deposited on a borosilicate glass substrate. The quantity of acetylacetone in the precursor solution had no significant effect on the optical and structural properties of the TiO_2_ thin films. Films consisted of the anatase phase, with a band gap in the range of 3.3–3.5 eV and thickness in the range of 280–410 nm. However, the morphological study of the TiO_2_ films showed that an increase in AcacH quantity in the precursor solution made the surface of the thin film more uniform.

Photocatalytic activity of the TiO_2_ thin films with different TTIP:AcacH molar ratios was studied by the stearic acid test under UV-A and VIS light. It was found that with the increase in AcacH in the precursor solution, the photodegradation rate of stearic acid increased rapidly under both irradiations. However, as expected, the photocatalytic activity of the films was much higher under UV-A. The film with TTIP:AcacH molar ratio 1:8 had the highest activity in SA degradation under UV-A with the reaction rate constant k = 0.2427 min^−1^, which is 10 times higher compared to the molar ratio 1:4. The film with the molar ratio of 1:8 also showed maximum activity under VIS with k = 0.0323 min^−1^, which is four times higher than for the molar ratio 1:4. The optimal molar ratio of TTIP:AcacH 1:8 for ultrasonically sprayed TiO_2_ thin films, which was found in this study, could be also applied for other chemical solution deposition techniques to produce photocatalytically efficient films.

The photodegradation rate of SA is enhanced with an increase in the AcacH amount in the precursor solution due to changes in the morphological and wettability properties and due to the incorporation of carbon into the TiO_2_ lattice. XPS results showed that the film with a higher AcacH molar ratio contains more adsorbed carbon (BE = 285 eV) on the surface of the film and more carbon incorporated into the lattice of TiO_2_, which presumably improves the photocatalytic activity of the films.

## Figures and Tables

**Table 1:** Optical properties of TiO_2_ thin films with different TTIP:AcacH ratios.

TTIP:AcacH Molar Ratio	Thickness, nm	Band Gap, eV
1:3	380	3.3
1:4	370	3.4
1:5	370	3.4
1:6	410	3.4
1:7	370	3.4
1:8	375	3.4
1:10	370	3.4
1:12	390	3.4
1:20	280	3.5

**Table 2:** XPS data of the TiO_2_ thin films after UV-A pre-treatment.

TTIP:AcacH Molar Ratio	(Vo)/(Ti-O) (at%/at%)	(OH^–^)/(Ti-O) (at%/at%)	(C-O)/(C-H) (at%/at%)	(Ti-O-C)/(C-H) (at%/at%)	(C=C)/(C-H) (at%/at%)
**1:4**	0.08	0.07	0.8	0.38	5.43
**1:8**	0.06	0.07	1.35	0.44	7.80

**Table 3:** Water contact angle values of as-deposited, one-month aged, and UV-A pre-treated films.

TTIP:AcacH Molar Ratio	Contact Angle°		
	As-Deposited	One-month Aged	UV-A 15 min
1:4	20	55	4
1:8	20	30	0
